# Comparative Evaluation of Photobiomodulation and Intracanal Cryotherapy on Post-Endodontic Pain Following Single-Visit Root Canal Treatment: A Randomized Controlled Trial

**DOI:** 10.7759/cureus.112182

**Published:** 2026-07-06

**Authors:** Krupali D Kalena, Shivangi M Pujara, Hardik B Shah, Leena H Jobanputra, Bhargav R Patel, Anupama Abhilasha Murmu

**Affiliations:** 1 Dentistry, GMERS (Gujarat Medical Education and Research Society) Medical College, Sola, Ahmedabad, IND; 2 Dentistry, Dantam Dental Clinic, Ahmedabad, IND; 3 Dentistry, Navdant Dental Clinic, Bhavnagar, IND; 4 Conservative Dentistry and Endodontics, Government Dental College and Hospital, Jamnagar, IND; 5 Community Medicine, Netaji Subhas Medical College and Hospital, Adityapur, Jamshedpur, IND

**Keywords:** intracanal cryotherapy, photobiomodulation, post-endodontic pain, randomized controlled trial, root canal treatment, visual analog scale

## Abstract

Background

Postoperative pain following root canal treatment (RCT) remains a clinically relevant concern despite continuing refinements in endodontic technique. This randomized controlled trial aimed to evaluate and compare the analgesic efficacy of photobiomodulation therapy (PBMT) and intracanal cryotherapy in reducing post-endodontic pain following a single-visit RCT.

Methods

Ninety adults requiring RCT of an asymptomatic mandibular first molar were allocated by computer-generated randomization into three parallel groups (n = 30 each): Group 1 (Control), final irrigation with 20 mL of room-temperature saline; Group 2 (PBMT), final saline irrigation followed by intraoral transmucosal 940 nm diode laser irradiation at 0.1 W for 40 s at each of four mucosal points (4 J per point; 16 J total per session); and Group 3 (Cryotherapy), final irrigation with 20 mL of chilled saline (2°C-4°C). Postoperative pain was assessed using a visual analog scale (VAS) at 6, 24, and 48 hours and at 7 days, with parallel recording of rescue analgesic intake. Intergroup comparisons of the non-normally distributed pain and analgesic data used the Kruskal-Wallis test; categorical data were compared by the chi-square test (α = 0.05).

Results

No statistically significant intergroup differences in VAS pain scores or analgesic intake were observed at any time point (p > 0.05). The cryotherapy group recorded the lowest mean pain score at six hours (0.53 ± 0.90) and the PBMT group at 24 hours (0.33 ± 0.76). All three groups reached a VAS of 0 by day 7, and no analgesic was consumed in any group at day 7.

Conclusions

Within the limits of this trial, neither PBMT nor intracanal cryotherapy conferred a statistically significant analgesic advantage over conventional saline irrigation following single-visit RCT in asymptomatic patients. As this population had minimal preoperative pain, the result should not be extrapolated to symptomatic patients, in whom adjunctive therapies may still be beneficial. This indicates that, in asymptomatic cases, meticulous canal disinfection and adherence to fundamental endodontic principles remain the principal determinants of postoperative outcomes.

## Introduction

Root canal treatment (RCT) is the definitive procedure for eliminating pulpal infection and preserving natural dentition. Its primary objective is the thorough debridement and disinfection of the root canal system through a combination of mechanical instrumentation, chemical irrigation, and three-dimensional obturation. Despite continual technical refinement, postoperative pain remains one of the most frequent patient complaints, with reported prevalence ranging from 1.5% to 53% across published series [1,2].

Single-visit RCT has gained widespread acceptance owing to patient convenience, fewer appointments, and clinical outcomes comparable with multivisit approaches [3]. However, the absence of an inter-appointment calcium hydroxide dressing in single-visit cases has raised theoretical concerns about residual periapical inflammation contributing to postoperative discomfort.

Post-endodontic pain is multifactorial in origin and is attributable to periapical tissue injury, extrusion of irrigant or debris, persistent microorganisms, and the consequent release of inflammatory mediators such as prostaglandins, bradykinin, and substance P. Inadequate working length control, over-instrumentation, and suboptimal irrigation are modifiable operator-dependent factors that significantly influence pain severity [4,5].

Management of post-RCT pain encompasses pharmacological strategies, such as non-steroidal anti-inflammatory drugs, acetaminophen, and corticosteroids, and non-pharmacological adjuncts. Among the latter, intracanal cryotherapy and low-level laser therapy or photobiomodulation therapy (PBMT) have attracted considerable research interest as adjuncts to routine endodontic care [6-8].

Cryotherapy delivered as a final cold saline irrigant (2°C-4°C) has been shown to reduce the external root-surface temperature by more than 10°C for up to four minutes [9]. This localized temperature reduction decreases enzymatic activity, limits the release of inflammatory mediators, attenuates periapical edema, and slows peripheral nerve conduction, thereby attenuating the pain response [10-12].

PBMT employs non-ionizing photonic energy at wavelengths in the red and near-infrared spectrum (approximately 600-1100 nm) to modulate cellular metabolism through stimulation of mitochondrial cytochrome c oxidase, increasing ATP synthesis and reducing oxidative stress [13,14]. Clinically, PBMT exerts anti-inflammatory, analgesic, and biomodulatory effects without producing significant thermal change in irradiated tissues [14]. Evidence from in vitro and in vivo investigations suggests that PBMT reduces nociceptor activity through local conduction blockade and targeted inhibition of inflammatory signaling pathways [13,15].

Despite the mechanistic plausibility of both modalities, prospective randomized data that directly compare them in managing post-endodontic pain remain scarce. The present study was therefore designed to evaluate and compare the analgesic effect of PBMT and intracanal cryotherapy following a single-visit RCT, with conventional saline irrigation serving as the control.

The null hypothesis stated that there would be no significant difference in postoperative pain or analgesic intake among the three groups at any follow-up interval.

## Materials and methods

Study design, setting, and ethical approval

This was a parallel-group, single-center, randomized controlled trial conducted in the Department of Conservative Dentistry and Endodontics, Government Dental College and Hospital, Jamnagar, Gujarat, India, between March 15, 2024, and February 28, 2025. The trial was reported in accordance with the CONSORT 2010 statement for parallel-group randomized trials [16]. The protocol was approved by the Institutional Ethics Committee of M.P. Shah Government Medical College and Guru Gobind Singh General Hospital, Jamnagar, India (which serves as the central IRB for all teaching units on the campus, including the Government Dental College and Hospital) under approval number 206/03/2023 and was prospectively registered with the Clinical Trials Registry - India (CTRI/2024/03/063495) before enrolment. All procedures were conducted in accordance with the principles of the Declaration of Helsinki. Written informed consent was obtained from every participant after a thorough explanation of the study objectives, procedures, and the voluntary nature of participation.

Sample size

Sample size was calculated a priori using G*Power software version 3.1.9.7 (Heinrich-Heine-Universität Düsseldorf, Düsseldorf, Germany) for a one-way analysis of variance (ANOVA) with three groups, assuming a large effect size (f = 0.40), a two-tailed alpha of 0.05, and statistical power (1 − β) of 0.95; this yielded a minimum requirement of 84 participants. To accommodate potential attrition, 90 participants (30 per group) were enrolled.

Participant selection

Participants were recruited from adult patients attending the outpatient clinic of the Department of Conservative Dentistry and Endodontics who required an RCT of a mandibular first molar. Inclusion criteria were age 18-60 years, fully developed roots, pulpal diagnosis of asymptomatic irreversible pulpitis or pulp necrosis, periapical diagnosis of normal periapical tissue or asymptomatic apical periodontitis, and a preoperative visual analog scale (VAS) score of 0. Exclusion criteria were symptomatic preoperative pain; systemic disease (uncontrolled diabetes mellitus and immunocompromised status); pregnancy or lactation; clinical history of swelling, sinus tract, or fistula; retreatment cases; root resorption; open apex; and analgesic intake within 12 hours preceding the procedure. Pulpal and periapical diagnoses were established based on both clinical evaluation (visual inspection, percussion, and palpation as well as thermal and electric pulp sensibility testing) and periapical radiographic examination.

Randomization and blinding

Participants were assigned to one of three parallel groups in a 1:1:1 ratio using a computer-generated allocation sequence prepared by a statistician not otherwise involved in the trial. The sequence was concealed in sequentially numbered, sealed, opaque envelopes that were opened only at the point of final irrigation. The outcome assessor scoring the postoperative VAS data was blinded to group allocation throughout the follow-up period. Owing to the visible nature of the interventions, operator blinding was not feasible.

Standard endodontic protocol

All procedures were performed by a single calibrated operator under rubber-dam isolation. A preoperative periapical radiograph was obtained for standardization using a radiovisiograph (RVG 5200, Carestream Health Inc., Atlanta, GA). Pulp sensitivity was assessed using an electric pulp tester (Parkell Digitest II, Parkell Inc., Edgewood, NY) and cold spray (Endo-Frost, Coltène/Whaledent AG, Altstätten, Switzerland). Local anesthesia was achieved with 2% lignocaine and 1:100000 adrenaline (Lignox 2% A, Indoco Remedies Ltd., Mumbai, India), administered as an inferior alveolar nerve block.

Access cavity preparation was performed using a high-speed air-rotor handpiece at 100,000 rpm with a safe-ended round cutting bur. Where indicated, pre-endodontic build-up was completed with direct composite resin (Filtek Z250, 3M ESPE, St. Paul, MN). Coronal pre-flaring was performed with a ProTaper Gold SX file (Dentsply Sirona Endodontics, Ballaigues, Switzerland). The working length was established using the Root ZX Mini apex locator (J. Morita Mfg. Corp., Kyoto, Japan) and confirmed by a periapical radiograph. Canal preparation was completed with ProTaper Next rotary nickel-titanium files (Dentsply Sirona Endodontics, Ballaigues, Switzerland; sizes X2-X5), with apical preparation maintained three sizes larger than the first binding file and a minimum apical diameter of ISO 25.

Irrigation was performed throughout instrumentation through a 30-gauge double-side-vented needle (NaviTip, Ultradent Products Inc., South Jordan, UT) positioned 1-2 mm short of the working length, delivering 2 mL of 3% sodium hypochlorite (Prime Dental Products Pvt. Ltd., Thane, India) per canal per file. Following completion of instrumentation, the canals were irrigated with 3% sodium hypochlorite activated using the EndoActivator system (Dentsply Sirona Endodontics, Tulsa, OK) for 60 seconds, followed by a normal-saline flush and 17% EDTA (Prevest DenPro Ltd., Jammu, India) for 60 seconds to remove the smear layer.

Intervention groups

Group 1 (Control)

Participants received final irrigation with 20 mL of normal saline at room temperature (20°C-22°C) delivered over five minutes.

Group 2 (PBMT)

**Figure 1:**
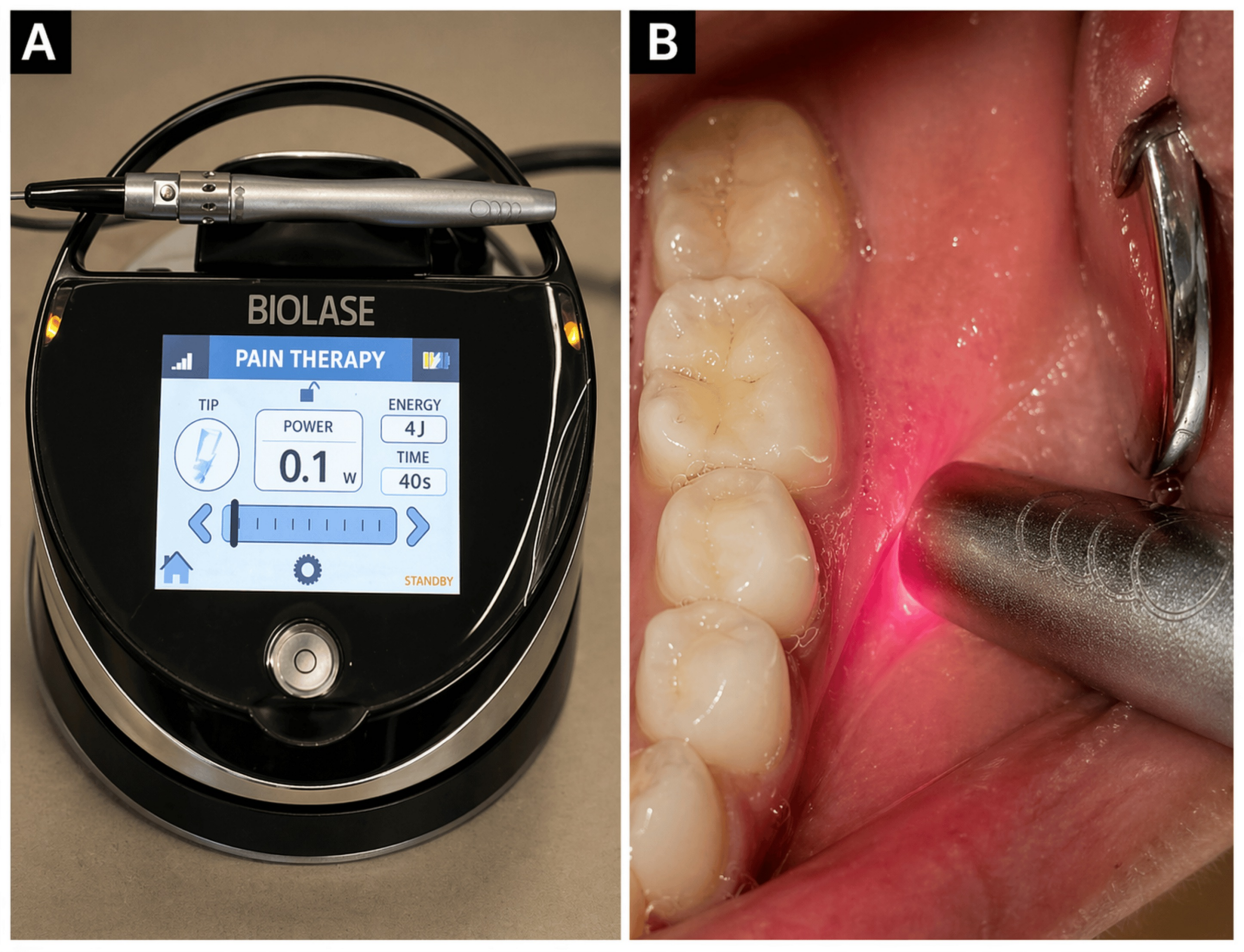
Photobiomodulation setup (A) Diode laser device (Epic X, Biolase Inc., Foothill Ranch, CA; 940 nm) displaying the treatment parameters per point (power, 0.1 W; energy, 4 J; time, 40 s). Each of the four points received 4 J, yielding 16 J total per session. (B) Application of laser irradiation at the buccal mucosal contact points in the photobiomodulation group.

Participants received final irrigation with 20 mL of normal saline over five minutes, followed immediately by intraoral photobiomodulation using a 940 nm diode laser (Epic X, Biolase Inc., Foothill Ranch, CA) in continuous wave mode at 0.1 W. Irradiation was applied with the handpiece tip in light contact with the mucosa for 40 seconds at each of four points (two buccal and two lingual). With the device operating at 0.1 W, each 40-second exposure delivered 4 J of energy per point (energy density, 8 J/cm² per point), giving a cumulative dose of 16 J per session over the four points (Figure 1).

Group 3 (Cryotherapy)

**Figure 2:**
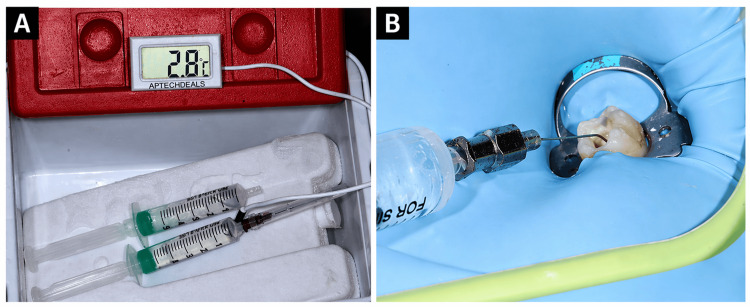
Cryotherapy setup (A) Calibrated digital thermometer confirming chilled saline temperature at 2°C-4°C for the cryotherapy group. (B) Application of chilled saline as the final irrigant via a 30-gauge double-sided-vented needle.

Participants received final irrigation with 20 mL of chilled normal saline (2°C-4°C) delivered over five minutes. Saline temperature was verified immediately before irrigation with a calibrated digital thermometer (HTC Instruments, Mumbai, India) and maintained throughout the procedure using an insulated ice bath (Figure 2).

Obturation was performed in all groups using gutta-percha (Dentsply Sirona Endodontics, Ballaigues, Switzerland) with AH Plus resin-based sealer (Dentsply DeTrey GmbH, Konstanz, Germany) by the cold lateral condensation technique. Post-endodontic coronal restoration was completed with direct composite resin (Filtek Z250, 3M ESPE, St. Paul, MN).

Outcome measures

The primary outcome was postoperative pain intensity measured using a 100 mm VAS, a sensitive instrument for measuring pain intensity [17], anchored at 0 mm (no pain) and 100 mm (worst imaginable pain), and recorded to the nearest centimeter (0-10 cm). Scores were interpreted using established cutoffs as no pain (0-4 mm), mild (5-44 mm), moderate (45-74 mm), or severe (75-100 mm) pain at 6, 24, and 48 hours and 7 days after treatment. The secondary outcome was rescue analgesic intake, recorded as the number of ibuprofen 400 mg tablets consumed during the same follow-up intervals. Participants experiencing pain were instructed to take ibuprofen 400 mg as required and to record each dose. All outcome measures were self-reported by participants using a structured diary provided at discharge. Participants recorded their pain scores independently at the specified time points, without the treating operator present; the completed diaries were collected and collated by an assessor blinded to group allocation, so operator presence did not influence VAS scoring.

Statistical analysis

All data were entered and verified in Microsoft Excel 2019 (Microsoft Corp., Redmond, WA) and analyzed using IBM SPSS Statistics for Windows, version 19.0 (IBM Corp., Armonk, NY). Continuous variables were summarized as mean ± standard deviation and categorical variables as frequency (percentage). The normality of the pain and analgesic-intake data was examined using the Shapiro-Wilk test; as these outcomes were non-normally distributed, intergroup comparisons were performed using the Kruskal-Wallis test, with Dunn’s test (Bonferroni correction) reserved for post-hoc pairwise comparison where the omnibus test was significant. Epsilon-squared (ε²) was reported as the effect-size measure, and 95% confidence intervals for between-group differences were obtained by bootstrap resampling. Age was compared using one-way ANOVA, and categorical demographic variables were compared with the chi-square test. A two-tailed p-value < 0.05 was considered statistically significant.

Participant flow

**Figure 3:**
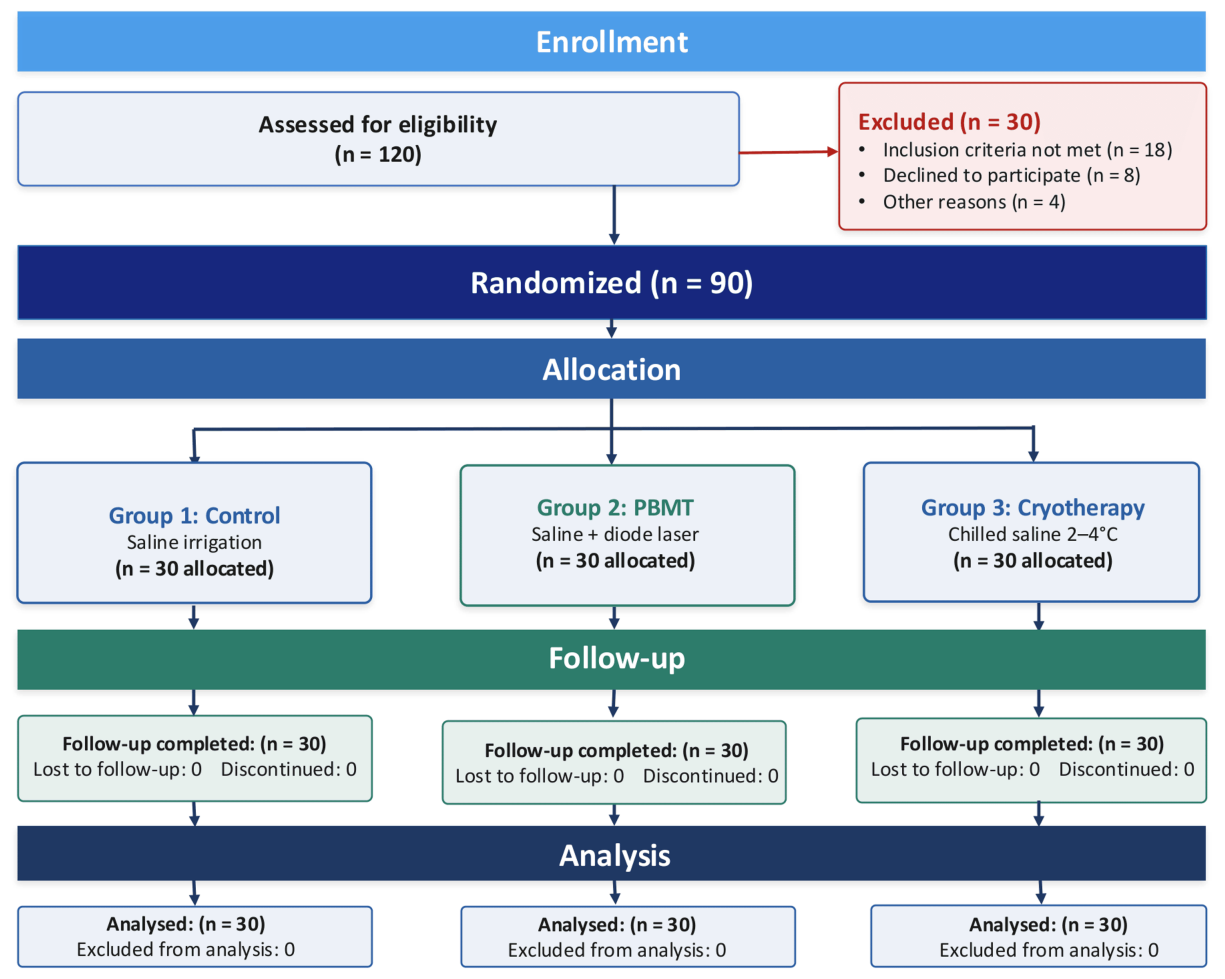
CONSORT 2010 flow diagram illustrating participant enrolment, allocation, follow-up, and analysis PBMT: Photobiomodulation therapy.

The flow of participants through each phase of the trial is presented in Figure 3. Of 120 patients assessed for eligibility, 30 were excluded (18 did not meet the inclusion criteria, eight declined to participate, and four were excluded for other reasons). All 90 enrolled participants completed the study, with no loss to follow-up or withdrawal in any group.

## Results

Ninety participants contributing 90 mandibular first molars were enrolled and analyzed (30 per group). All participants had a preoperative VAS score of 0 and had not taken any analgesics within 12 hours of the procedure. No participant was lost to follow-up, and all 90 were included in the per-protocol analysis.

Demographic and baseline clinical characteristics

The three groups were comparable at baseline with respect to age, sex distribution, tooth side, and pulpal and periapical diagnoses (all p > 0.05; Table 1).

**Table 1:** Demographic and clinical characteristics of participants across the three study groups Values are expressed as mean ± standard deviation (SD) for continuous variables and as frequencies (n) for categorical variables. P-values were obtained using one-way analysis of variance (ANOVA) for continuous data and the chi-square (χ²) test for categorical data. PBMT: Photobiomodulation therapy.

Parameters	Control (n = 30)	PBMT (n = 30)	Cryotherapy (n = 30)	P-value	Test
Age (years), mean ± SD	32.4 ± 9.1	31.8 ± 9.4	33.1 ± 8.7	0.86	ANOVA
Sex					
Male, n (%)	14 (46.7%)	15 (50.0%)	16 (53.3%)	0.87	χ²
Female, n (%)	16 (53.3%)	15 (50.0%)	14 (46.7%)		
Tooth side					
Right, n (%)	16 (53.3%)	17 (56.7%)	15 (50.0%)	0.83	χ²
Left, n (%)	14 (46.7%)	13 (43.3%)	15 (50.0%)		
Pulpal diagnosis					
Asymptomatic irreversible pulpitis, n (%)	18 (60.0%)	19 (63.3%)	17 (56.7%)	0.84	χ²
Pulp necrosis, n (%)	12 (40.0%)	11 (36.7%)	13 (43.3%)		
Periapical diagnosis					
Normal, n (%)	20 (66.7%)	22 (73.3%)	21 (70.0%)	0.79	χ²
Asymptomatic apical periodontitis, n (%)	10 (33.3%)	8 (26.7%)	9 (30.0%)		

VAS pain scores

Mean VAS pain scores at each time interval are summarized in Table 2. At six hours postoperatively, the cryotherapy group recorded the lowest mean pain score (0.53 ± 0.90), followed by the PBMT group (0.60 ± 0.93) and the control group (0.70 ± 1.12). At 24 hours, the PBMT group recorded the lowest mean score (0.33 ± 0.76), followed by cryotherapy (0.40 ± 0.81) and control (0.43 ± 0.90). At 48 hours, both the cryotherapy and control groups recorded a mean of 0.00 ± 0.00, while the PBMT group recorded a mean of 0.07 ± 0.37, attributable to a single participant. All three groups achieved a mean VAS score of 0.00 ± 0.00 at seven days. Despite these numerical trends, the Kruskal-Wallis test detected no statistically significant intergroup differences at any time point (6 h: H = 0.27, p = 0.875; 24 h: H = 0.17, p = 0.918; 48 h: H = 2.00, p = 0.368), with negligible-to-small effect sizes (ε² = 0.003, 0.002 and 0.022, respectively) and 95% confidence intervals for the between-group differences that all included zero.

**Table 2:** Postoperative pain scores on the visual analog scale across groups and time points. Values are expressed as mean ± standard deviation (SD) on a 0–10 visual analog scale. Because the data were non-normally distributed (Shapiro-Wilk P < 0.001, reflecting a floor effect), intergroup comparisons were performed using the Kruskal-Wallis test. The H statistic, P-value, and epsilon-squared (ε²) effect size are reported. As the test was nonsignificant at every interval, no post-hoc comparisons were performed, and the median score was 0 at all time points. VAS: Visual analog scale; PBMT: Photobiomodulation therapy.

Group	Pre-op	6 hours	24 hours	48 hours	7 days
Control (n = 30)	0.00 ± 0.00	0.70 ± 1.12	0.43 ± 0.90	0.00 ± 0.00	0.00 ± 0.00
PBMT (n = 30)	0.00 ± 0.00	0.60 ± 0.93	0.33 ± 0.76	0.07 ± 0.37	0.00 ± 0.00
Cryotherapy (n = 30)	0.00 ± 0.00	0.53 ± 0.90	0.40 ± 0.81	0.00 ± 0.00	0.00 ± 0.00
H-value	-	0.27	0.17	2.00	-
p-value	-	0.875	0.918	0.368	-
ε² (effect size)	-	0.003	0.002	0.022	-

Analgesic intake

Mean rescue analgesic intake (ibuprofen 400 mg tablets) across groups and time points is summarized in Table 3. At six hours, the cryotherapy group recorded the lowest mean intake (0.13 ± 0.35), followed by control (0.23 ± 0.43) and PBMT (0.27 ± 0.45). At 24 hours, the cryotherapy group again had the lowest mean intake (0.07 ± 0.25), followed by PBMT (0.13 ± 0.35) and control (0.13 ± 0.35). At 48 hours, both the cryotherapy and control groups reported zero analgesic use, whereas the PBMT group showed minimal residual intake (0.03 ± 0.18). No analgesic intake was recorded in any group at seven days. The Kruskal-Wallis test detected no statistically significant intergroup differences at any time point (6 h: H = 1.72, p = 0.424; 24 h: H = 0.89, p = 0.641; 48 h: H = 2.00, p = 0.368).

**Table 3:** Postoperative rescue analgesic intake across groups and time points Values are expressed as mean ± standard deviation (SD) of 400 mg ibuprofen tablets consumed during each interval. Intergroup comparisons were performed using the Kruskal-Wallis test. The H-statistic, P-value, and epsilon-squared (ε²) effect size are reported. PBMT: Photobiomodulation therapy.

Group	Pre-op	6 hours	24 hours	48 hours	7 days
Control (n = 30)	0.00 ± 0.00	0.23 ± 0.43	0.13 ± 0.35	0.00 ± 0.00	0.00 ± 0.00
PBMT (n = 30)	0.00 ± 0.00	0.27 ± 0.45	0.13 ± 0.35	0.03 ± 0.18	0.00 ± 0.00
Cryotherapy (n = 30)	0.00 ± 0.00	0.13 ± 0.35	0.07 ± 0.25	0.00 ± 0.00	0.00 ± 0.00
H-value	-	1.72	0.89	2.00	-
p-value	-	0.424	0.641	0.368	-
ε² (effect size)	-	0.019	0.010	0.022	-

## Discussion

The principal finding of this randomized controlled trial is that neither PBMT nor intracanal cryotherapy demonstrated a statistically significant reduction in postoperative pain or rescue analgesic intake compared with conventional saline irrigation following single-visit RCT in asymptomatic patients. These results are consistent with the null hypothesis and corroborate an emerging body of evidence indicating that the quality of canal preparation and disinfection is the dominant determinant of postoperative outcomes.

The low overall pain burden observed across all three groups, with approximately 70% of patients reporting no pain at any time point and requiring no rescue analgesia, likely reflects the stringent inclusion criteria (asymptomatic patients with a preoperative VAS of 0) and the standardized, high-quality endodontic protocol employed. It is well established that preoperative pain is the strongest predictor of postoperative pain [18,19]; enrolling only asymptomatic patients inherently reduces the baseline inflammatory load and diminishes the opportunity for adjunctive interventions to demonstrate superiority [16,20].

The observed numerical trend toward lower pain scores in the cryotherapy group at six hours is mechanistically plausible. Intracanal cryotherapy using cold saline (2°C-4°C) lowers the external root-surface temperature by more than 10°C for up to four minutes [9], suppressing periapical inflammatory mediator release, reducing edema, and slowing A-δ and C fiber nociceptor conduction [10,11]. Both Keskin et al. [21] and Vera et al. [12] previously reported significant reductions in postoperative pain at 6 and 24 hours with intracanal cryotherapy compared with saline controls, and the systematic review by Sadaf et al. [8] reached a similar pooled conclusion. The failure to reach statistical significance in the present study likely reflects the floor effect imposed by uniformly low preoperative pain scores and the relatively small sample per group.

It is important to distinguish the population studied here from those of the trials that reported a positive effect. Keskin et al. [21] and Vera et al. [12] enrolled patients with symptomatic, often acutely inflamed or necrotic teeth, in whom baseline pain and inflammatory burden are high, therefore demonstrating substantial pain reduction.The present trial, by contrast, enrolled only asymptomatic teeth with a preoperative VAS of 0, in whom postoperative pain is infrequent and generally mild. A null result in this setting is the expected outcome rather than a contradiction of the earlier work: the two bodies of evidence address different clinical questions - whether an adjunctive therapy relieves existing symptomatic pain and whether it prevents pain in patients who are already pain-free. Because the scope for additional benefit in a low-pain, asymptomatic population is intrinsically narrow, the capacity of any adjunctive measure to outperform effective canal disinfection alone is correspondingly limited.

The transient trend toward lower scores in the PBMT group at 24 hours is also mechanistically consistent. Photobiomodulation at 940 nm activates cytochrome c oxidase in the mitochondrial electron-transport chain, increasing ATP production, reducing reactive oxygen species, and modulating nuclear factor - kappa B - a key transcription factor in inflammatory signaling [13,14]. These cellular events typically manifest with a latency of 12-24 hours [13,15], consistent with the delayed analgesic response observed in this study compared with cryotherapy. Randomized trials by Naseri et al. [22] and Angolkar et al. [23] have demonstrated significant post-endodontic pain reduction with PBMT compared with placebo at 24 and 48 hours. However, patient profiles and laser parameters varied considerably across these trials.

The marginally higher mean VAS score within the PBMT group at 48 hours (0.07 ± 0.37) was attributable to a single participant and is unlikely to be clinically meaningful. Should a comparable pattern prove reproducible in larger samples, one speculative explanation would be the biphasic dose-response relationship characteristic of PBMT - the so-called Arndt-Schultz principle - by which sub-therapeutic or supra-therapeutic dosing may paradoxically stimulate rather than inhibit inflammatory pathways [13,24]. The total energy delivered (16 J distributed across four mucosal points; 4 J per point) lay within the recommended therapeutic window described in the literature [15,24]; however, individual variation in tissue absorption, skin pigmentation, and mucosal thickness may have altered the effective fluence reaching the periapical target tissue [13,15]. The intraoral transmucosal delivery employed in this study may also have reduced the energy reaching the periapical environment compared with intracanal delivery systems - an issue that warrants direct investigation in future work.

A further consideration concerns the relationship between the interventions and the assessment schedule. Both cryotherapy and photobiomodulation were delivered as a single, brief application whose direct physiological effects - local vasoconstriction and slowed nerve conduction for cryotherapy as well as transient photonic modulation of cellular metabolism for PBMT - are expected to last minutes to hours rather than days. The rationale for assessing pain at 48 hours and 7 days was not to determine whether these immediate effects persisted, but to evaluate whether attenuation of the acute inflammatory response induced by instrumentation reduced the magnitude and duration of subsequent inflammatory pain. It remains mechanistically unlikely, however, that a single short-acting application would exert a measurable influence at these later intervals, and this temporal mismatch is itself a plausible contributor to the absence of any between-group difference beyond the immediate postoperative period. A durable effect, if attainable at all, would more plausibly require repeated applications timed to the evolving inflammatory response - an approach that warrants evaluation in future trials.

From a clinical perspective, the present findings suggest that, in carefully selected asymptomatic patients treated with meticulous single-visit RCT, the addition of PBMT or cryotherapy as adjuncts does not provide measurable analgesic benefit over an optimized irrigation protocol alone. Given that post-endodontic pain prevalence is consistently lower in well-controlled, asymptomatic cohorts [1,25], the marginal absolute benefit available to any adjunctive therapy in such populations is intrinsically constrained. These observations have cost, time, and chairside-efficiency implications for routine clinical practice [3,25]. However, the conclusions should not be extrapolated to symptomatic cases, retreatment cases, or patients with established periapical pathology, in whom the inflammatory milieu is substantially different and adjunctive therapies may yield greater benefit [8,21].

Strengths and limitations

The strengths of this study include prospective registration, standardized intervention protocols, a blinded outcome assessor, complete follow-up with zero attrition, and a comprehensive assessment schedule spanning seven days. All procedures were performed by a single calibrated operator, thereby eliminating inter-operator variability.

Limitations include the single-center design, which constrains external generalizability. Operator blinding was not feasible because of the nature of the interventions, introducing potential performance bias. The study was restricted to asymptomatic patients, which limits its applicability to symptomatic presentations. The seven-day follow-up did not capture long-term outcomes. The PBMT was applied via intraoral transmucosal irradiation rather than intracanally; intracanal delivery might achieve more direct periapical tissue effects. Finally, the study was not powered to detect small effect sizes, and a larger sample may have revealed statistically - if not clinically - meaningful differences between groups.

Future research should evaluate these modalities in symptomatic patients with pre-existing periapical pathology, investigate intracanal laser delivery systems, explore combination approaches (simultaneous cryotherapy and PBMT), and incorporate objective biomarker outcomes such as periapical cytokine levels.

## Conclusions

Within the limits of this randomized controlled trial, neither photobiomodulation therapy nor intracanal cryotherapy demonstrated a statistically significant advantage over conventional saline irrigation in reducing postoperative pain or rescue analgesic intake following a single-visit RCT in asymptomatic patients. The uniformly low pain burden across all three groups underscores that, in asymptomatic cases, thorough canal disinfection and adherence to fundamental endodontic principles remain the principal determinants of postoperative outcomes. This result reflects the absence of a detectable difference in asymptomatic teeth specifically and should not be interpreted as an absence of effect in endodontic patients generally. In particular, it should not be extrapolated to symptomatic patients, for whom prior evidence indicates a benefit from these adjuncts. These adjunctive modalities may warrant further investigation in symptomatic patient populations and in study designs that account for the floor effects inherent in asymptomatic cohorts.
